# Context factors in clinical decision-making: a scoping review

**DOI:** 10.1186/s12911-025-02965-1

**Published:** 2025-03-17

**Authors:** Katharina Schuler, Ian-C. Jung, Maria Zerlik, Waldemar Hahn, Martin Sedlmayr, Brita Sedlmayr

**Affiliations:** 1 Institute for Medical Informatics and Biometry, Faculty of Medicine, University Hospital Carl Gustav Carus, TUD Dresden University of Technology, Fetscherstraße 74, 01307 Dresden, Germany; 2 Center for Scalable Data Analytics and Artificial Intelligence (ScaDS.AI), Dresden/Leipzig, Dresden, Germany

**Keywords:** Clinical decision-making, Clinical decision support systems, Review, Patient-centered care, Context factors

## Abstract

**Background:**

Clinical decision support systems (CDSS) frequently exhibit insufficient contextual adaptation, diminishing user engagement. To enhance the sensitivity of CDSS to contextual conditions, it is crucial first to develop a comprehensive understanding of the context factors influencing the clinical decision-making process. Therefore, this study aims to systematically identify and provide an extensive overview of contextual factors affecting clinical decision-making from the literature, enabling their consideration in the future implementation of CDSS.

**Methods:**

A scoping review was conducted following the PRISMA-ScR guidelines to identify context factors in the clinical decision-making process. Searches were performed across nine databases: PubMed, APA PsycInfo, APA PsyArticles, PSYINDEX, CINAHL, Scopus, Embase, Web of Science, and LIVIVO. The search strategy focused on combined terms related to contextual factors and clinical decision-making. Included articles were original research articles written in English or German that involved empirical investigations related to clinical decision-making. The identified context factors were categorized using the card sorting method to ensure accurate classification.

**Results:**

The data synthesis included 84 publications, from which 946 context factors were extracted. These factors were assigned to six primary entities through card sorting: patient, physician, patient’s family, institution, colleagues, and disease treatment. The majority of the identified context factors pertained to individual characteristics of the patient, such as health status and demographic attributes, as well as individual characteristics of the physician, including demographic data, skills, and knowledge.

**Conclusion:**

This study provides a comprehensive overview of context factors in clinical decision-making previously investigated in the literature, highlighting the complexity and diversity of contextual influences on the decision-making process. By offering a detailed foundation of identified context factors, this study paves the way for future research to develop more effective, context-sensitive CDSS, enhancing personalized medicine and optimizing clinical outcomes with implications for practice and policy.

## Introduction

Despite continuous advancements in knowledge about effective medical therapies, the implementation in clinical practice lags behind. Generating increasingly larger datasets makes it progressively more challenging for physicians to derive evidence-based recommendations from the ever-expanding information [1]. It often takes several years for new guidelines to be applied in routine practice, leading to a discrepancy between knowledge and practice, which can jeopardize patient safety [2]. Clinical decision support systems (CDSS) have the potential to bridge this gap and improve the quality of care by assisting physicians in the clinical decision-making process [3, 4]. Clinical decision-making is a complex, dynamic, and context-dependent process in which data are collected, interpreted, and evaluated to select an evidence-based course of action from several alternatives [5, 6]. Clinical decisions, which encompass diagnosis, monitoring, and interventions [7], often need to be made under conditions of uncertainty, as not all relevant information is fully available [6]. Furthermore, patient-specific variables such as genetic factors, medical history, or individual preferences further complicate the decision-making process. In such cases, clinical decision support systems (CDSS) can be of critical value, as they consolidate information from medical and patient-related sources, providing clinicians with targeted clinical data and evidence-based recommendations to optimize the decision-making process. For example, adherence to clinical guidelines can be promoted through automatic reminders and system recommendations, or early intervention can be facilitated by warnings about adverse drug interactions [4]. In this way, CDSS have the potential to significantly enhance the quality of clinical decisions by analyzing large datasets and providing relevant information. However, the effectiveness can vary considerably if the systems are not tailored to the specific context of use [4]. Insufficient adaptation to the workflows and requirements of the usage context can significantly impair the systems’ efficiency and acceptance [8]. Furthermore, inadequate consideration of specific patient characteristics or preferences can adversely affect patient care [8]. Therefore, to optimally support clinical decision-making, it is essential to sensitize the systems to contextual conditions to increase their effectiveness. Context-sensitive CDSS can provide personalized and precise recommendations that are not only based on clinical guidelines but also take into account the specific conditions and needs of the individual patient and the unique circumstances of the treatment situation [9, 10]. For the systems to optimally support the clinical decision-making process, it is necessary to present the right information to the right person at the right time in the workflow [11]. Adaptability to contextual conditions is thus a prerequisite for effectively using the systems [11]. To achieve this adaptability in practice, a user-centered design (UCD) approach [12, 13] is essential, as it systematically incorporates the needs, preferences, and usage contexts of the end users. A thorough analysis of users, their tasks, and their respective usage environments enables the precise identification and integration of relevant contextual factors into the design. In this way, CDSS are not only technically advanced, but also effectively usable and accepted in clinical practice. Nonetheless, despite established design principles [12, 13], the potential of context-based information in current CDSS remains largely untapped [14], in part because the construct of context is often insufficiently addressed. Context is often characterized by synonyms such as environment [15] or situation [16] or is described through examples like temperature, time, or noise level [17]. This way of characterizing context is inadequate for the practical application of context information, as an operationalized approach is required to create a structured approach that provides both a natural understanding of the concept of context and an implementation possibility for developers. One approach is provided by the definition according to [18], where context is understood as the entirety of information that can be used to characterize an entity, which can be a person, place, or object [18]. Context factors describe the individual characteristics that, in sum, describe the situation of an entity [19]. Context factors can thus be understood as attributes of an entity and can be assigned to the main categories of individuality, activity, location, time, and relationship [20]. Individual factors describe all properties and attributes that characterize the entity itself, such as demographic data, abilities, or emotional states [20]. Activity-related factors include all tasks an entity may be involved in, encompassing physical and mental activities relevant to specific situations [20]. For example, a patient’s physical activities, such as regular jogging, fall into the activity category. Location-related characteristics describe the spatial coordinates of an entity and refer to the physical location where an entity is situated [20], such as the physician’s place of residence or work environment. All temporal aspects, such as the duration of an illness, the timing of a diagnosis, or the chronological sequence of symptoms, are context factors that can be defined as descriptive time information. Factors assigned to the category of relationships include information about the relationships of one entity to another [20], such as a physician’s collaboration with colleagues or a patient’s family ties. Various context factors have already been investigated in the literature. However, these studies often focus on specific clinical decisions, such as prescribing antidepressants [21], or have been conducted for decisions in specific medical specialties, such as oncology [22]. These findings cannot be readily transferred, as the influence of a factor is significantly dependent on the specific conditions and characteristics of the original environment. For example, preoperative optimization of anesthesia for planned operations in surgery leads to better postoperative outcomes [23]. In emergency medicine, however, rapid and improvised anesthesia is often required, as patients are presented in critical conditions and often with unknown medical histories [24]. Specific requirements, risk profiles, and practice standards thus limit the transferability of factors between different medical specialties and care forms. Contextual conditions and the availability of information are crucial for the quality of clinical decisions. To technologically support the clinical decision-making process optimally, a comprehensive analysis of potential influencing factors is first necessary. Previous works that have focused on systematically compiling context factors often concentrate, like the empirical studies on the factors themselves, on specific specialties, diseases, and treatments (e.g [25–30]). or specific context factors, such as the family history of the patient [31] or system factors [32]. In contrast, research that provides a more comprehensive picture of contextual influence factors is not based on a systematic approach [6] or does not specifically refer to the clinical decision-making process, but, for example, the quality of care [33]. The context construct is only limitedly considered in previous works and is, for example, restricted to patient and environment-related influencing factors [33, 34]. The necessity of a broader systematic investigation and aggregation of previously investigated context factors arises from the limitations of previous research. Such a systematic approach enables the transparent presentation of the diversity and scope of investigated context factors, providing a solid foundation for a deeper understanding of their relevance in clinical decision-making processes. This facilitates CDSS developers in systematically incorporating these factors into the development process and also identifies opportunities for future research. Context factors provide valuable insights into decision-relevant information that algorithms should consider. Thus, comprehensive and thorough data integration is crucial for determining in the future which systems already possess context-based information that can be utilized, and where such data needs to be newly collected (e.g., through additional questions during anamnesis or by implementing supplementary form fields) in order to be used by algorithms in the future. Therefore, this work aims to systematically compile the context factors of the clinical decision-making process that have been investigated in the literature to date, thereby establishing a foundation for further research and development of context-sensitive algorithms for CDSS. This study focuses on systematically compiling and categorizing context factors in the clinical decision-making process to provide a structured overview, however, it does not aim to assess specific implementation strategies or their applicability across different settings. Grounded in a user-centered design approach, this scoping review is designed to assists CDSS developers in identifying and compiling relevant contextual factors to enhance overall system effectiveness and feasibility of these systems.

### Research question

Given the limitations of prior research, this study seeks to systematically identify and compile context factors previously examined in clinical decision-making. This investigation is guided by the following central research questions (RQ):

#### RQ

What context factors have been examined in physicians’ clinical decision-making?

- To which entities can the identified context factors be attributed?
- To which context categories can the entity-specific context factors be classified?

## Method

A systematic scoping review was conducted to address the central research questions. The methodological approach of the study was primarily guided by the Joanna Briggs Institute (JBI) manual for evidence synthesis [35] while also incorporating relevant principles from the Cochrane Handbook for Systematic Reviews [36] to ensure methodological rigor and comprehensiveness. This approach included detailed procedures for literature search, selection criteria, data extraction, and synthesis. The review process was further structured according to the PRISMA-ScR (Preferred Reporting Items for Systematic Reviews and Meta-Analyses extension for Scoping Reviews) guidelines [37], which provided a robust framework for reporting and evaluating the scope of the research comprehensively and systematically.

### Identification of the research question

To identify the central search components and translate them into an appropriate search strategy, the main research question was reformulated into a searchable format according to the PCC (Population-Concept-Context) framework [38]. The central research question (RQ) was decomposed into the search components outlined in Table 1 in accordance with the PCC framework.

**Table 1:** PCC framework for identifying the main concepts of the review

PCC-Components	Domain
**P**opulation	Physicians
**C**oncept	Context factors influencing clinical Decision-making
**C**ontext	clinical environment

### Identification of relevant studies

The search was conducted in April 2023 across the following nine databases: PubMed, APA PsycInfo, APA PsyArticles PSYINDEX, CINAHL, Scopus, Embase, Web of Science, and LIVIVO. The central search components “context factors” and “clinical decision-making” were combined using the Boolean term AND. Synonyms for the central search components were identified through MeSH terms and manual searches and were subsequently linked using the Boolean operator OR. Table 2 provides an overview of the search terms used for each database. The search string was adjusted according to the syntax of each database. No filters were applied during the search process to maximize the retrieval of relevant studies. To enhance search precision, the search terms were limited to titles and abstracts. Publications identified through the database queries were then loaded into the Zotero Citation Management Program [39]. Subsequently, all identified duplicate publications were manually removed by KS.

**Table 2:** Database and search terms

Database	Search terms
PubMed	(“context* factor*“[Title/Abstract] OR “impact* factor*“[Title/Abstract] OR “factor* influenc*“[Title/Abstract] OR “factor* impact*“[Title/Abstract] OR (“influencing“[Title/Abstract] AND “factor*“[Title/Abstract])) AND (“clinical decision making“[Title/Abstract] OR “medical decision making“[Title/Abstract] OR “clinical reasoning“[Title/Abstract] OR “clinical judgement“[Title/Abstract])
APA PsycInfo* APA PsyArticles* Psycindex* CINAH*	TI ((“context* factor*” OR “impact* factor*” OR “factor* influenc*” OR “factor* impact*” OR (“influencing” AND “factor*”)) AND (“clinical decision making” OR “medical decision making” OR “clinical reasoning” OR “clinical judgement*”)) OR AB ((“context* factor*” OR “impact* factor*” OR “factor* influenc*” OR “factor* impact*” OR (“influencing” AND “factor*”)) AND (“clinical decision making” OR “medical decision making” OR “clinical reasoning” OR “clinical judgement*”))
Scopus	(TITLE-ABS(“context* factor*” OR “impact* factor*” OR “factor* influenc*” OR “factor* impact*” OR (“influencing” AND “factor*”))) AND (TITLE-ABS (“clinical decision making” OR “medical decision making” OR “clinical reasoning” OR “clinical judgement*”))
Embase	((context* factor* or impact* factor* or factor* influenc* or factor* impact* or (influencing and factor*)) and (clinical decision making or medical decision making or clinical reasoning or clinical judgement*)).ab. or ((context* factor* or impact* factor* or factor* influenc* or factor* impact* or (influencing and factor*)) and (clinical decision making or medical decision making or clinical reasoning or clinical judgement*)).ti.
Web of Science	(TI=((“context* factor*” OR “impact* factor*” OR “factor* influenc*” OR “factor* impact*” OR (“influencing” AND “factor*”)) AND (“clinical decision making” OR “medical decision making” OR “clinical reasoning” OR “clinical judgement*”) (Title) OR (“context* factor*” OR “impact* factor*” OR “factor* influenc*” OR “factor* impact*” OR (“influencing” AND “factor*”)) AND (“clinical decision making” OR “medical decision making” OR “clinical reasoning” OR “clinical judgement*”))) OR AB=((“context* factor*” OR “impact* factor*” OR “factor* influenc*” OR “factor* impact*” OR (“influencing” AND “factor*”)) AND (“clinical decision making” OR “medical decision making” OR “clinical reasoning” OR “clinical judgement*”) (Title) OR (“context* factor*” OR “impact* factor*” OR “factor* influenc*” OR “factor* impact*” OR (“influencing” AND “factor*”)) AND (“clinical decision making” OR “medical decision making” OR “clinical reasoning” OR “clinical judgement*”))

Note. *The databases APA PsycInfo, APA PsycInfo, APA PsycArticles, PSYINDEX, and CINAHL were queried via the EBSCOhost research platform using a comprehensive search term

### Eligibility criteria for source selection

Publications were selected in two phases, adhering to the four-eyes principle. Initially, a title-abstract screening (TAS) was performed, followed by a full-text screening (FTS). For both stages, the relevant publications were uploaded to the research collaboration platform Rayyan (Qatar Computing Research Institute and Cochrane Bahrain) [40], with separate folders established for TAS and FTS. Only publications that did not violate any exclusion criteria (Table 3) were included during both screening phases.

**Table 3:** Descriptions of inclusion and exclusion criteria

Reason	Inclusion Criteria	Exclusion Criteria
Language	The article is written in English or German.	The article is not written in English or German.
Article type	The article is an original research article.	The article is not an original research article.
Clinical decision making	The decision-making process described in the article refers to diagnostic, treatment, prevention, or monitoring decisions that relate to the patient.	The decision-making process described in the article does not refer to diagnostic, treatment, prevention, or monitoring decisions that relate to the patient.
Decision maker	The clinical decision is made by a physician or human medicine student during the internship (practical year)	The decision is not made by a physician or human medicine student during the internship (practical year).
Context factors	The context factors described in the article are explicitly named and relate to clinical decision-making and describe aspects that are expressed outside the physical boundaries of a patient and are *not* directly physiologically and/or anatomically related to the patient’s principal diagnosis and do *not* refer to the pharmacodynamics or pharmacokinetics effects of drugs, imaging procedures, or medical interventions that are directly related to the principal diagnosis and/or directly affect the patient’s physiology or anatomy.	The context factors described in the article are not explicitly named or do not relate to clinical decision-making or describe aspects that are not expressed outside the physical boundaries of a patient or are directly physiologically and/or anatomically related to the patient’s principal diagnosis, such as genetic factors, laboratory values, vital signs etc. or refer to the pharmacodynamics or pharmacokinetics effects of drugs, imaging procedures, or medical interventions that are directly related to the principal diagnosis and/or directly affect the patient’s physiology or anatomy.
Empirical research	The context factors described in the article were investigated through empirical research methods.	The context factors described in the article were not investigated by empirical research methods but are based, for example, on theoretical considerations, opinions etc.
Access	The full text of the article can be accessed by the authors.	The full text of the article cannot be accessed by the authors.

For both screening rounds (TAS/FTS), publications were divided into three units alphabetically by the first author’s last name. Each unit was independently screened by the corresponding author (KS) and one additional rater (MZ, IJ, WH). Both screening stages were conducted in Rayyan [40] using the blind mode, ensuring that the raters were unaware of each other’s decisions during the process. Upon completion of the screenings, the blind mode was deactivated, and any conflicts between raters were resolved through discussion. In cases where consensus could not be achieved, the publication was referred to a third screener, who made the final decision.

### Data extraction

The information considered for data extraction was defined through an a posteriori process, aiming to thoroughly address the research questions and comprehensively capture the included studies’ content. The data from the final included publications were entered into a Microsoft Excel spreadsheet containing the information outlined in Table 4 Two reviewers independently conducted the data extraction, similar to the screening process. The corresponding author and one of four authors (MZ, IJ, WH, BS) extracted information from all publications in collaboration with one of four additional reviewers (MZ, IJ, WH, BS). Upon completion of data extraction, the results were compared, and any conflicts were resolved through discussion.

**Table 4:** Categories for data extraction

Category	Data	Description
Metadata	title	title of publication
	author	name of authors
	year	year of publication
	country	country of publication
	article type	type of article
Study characteristics	research type	quantitative, qualitative, mixed-method
	site of study conduction	country where the study was conducted
Medical scope	care sector	inpatient, outpatient, both, NA
	subject area	the subject area of data collection
	type of decision	diagnose, monitoring, intervention, admission, management, CDM in general
Context factors	reported impact	reported impact of CF on CDM
	no reported impact	no reported impact of CF on CDM

Note. NA = not applicable; CF = context factors; CDM = clinical decision-making

### Methodological quality assessment of selected studies

To ensure the quality of all included studies, a comprehensive quality assessment was conducted for each included publication using the quality assessment tool developed by Hawker et al. [41]. This tool was selected because it allows evaluating of both quantitative and qualitative studies, ensuring a consistent methodological evaluation across all included articles. The instrument comprises nine questions on abstract and title, introduction and aims, methods and data, sampling, data analysis, results, transferability and generalizability, and implications and usefulness. Each question can be rated as “good,” “fair,” “poor,” or “very poor.” These ratings were converted into numerical scores for the assessment, with “very poor” coded as 1 and “good” coded as 4. The studies were evaluated by a team of two individuals, with the corresponding author (KS) and one additional author (MZ, IJ, WH, BS) independently assessing each publication.

### Clustering of context factors

The synthesis of context factors was performed utilizing the card sorting technique [42]. For this purpose, a card sorting workshop was conducted in November 2023. A detailed methodological description of the workshop and its results have been published separately [43]. The workshop consisted of three consecutive sessions, each lasting four hours. Participants included three researchers from the field of human-computer interaction and one research assistant from the field of computer science. The categorization process was guided by the context definition provided by Zimmermann et al. [20], encompassing individuality, time, relation, activity, and locational contexts. The context factors extracted from the literature were written verbatim on individual cards for the card sorting. Each card contained the extracted context factor and an identification code to facilitate the later assignment to the respective publication from which the context factor was derived. To ensure orderly categorization, the cards were displayed on a whiteboard. The card sorting process was executed in three distinct phases. The initial phase employed a closed card sorting method, characterized by providing predefined categories to participants, serving as the foundation for the categorization process [42]. In this phase, participants were tasked with defining an entity and then systematically assigning the identified context factors to the predefined context categories: individuality, time, relation, activity, and location. The second and third phases incorporated an open card sorting approach, wherein participants independently categorized and labeled the information without predefined categories [42]. In the second phase, participants were required to further subdivide the context factors (already allocated to the primary context categories of an entity) into additional subcategories through consensus. The final phase involved the naming of the newly formed subcategories. Here, participants engaged in a consensus-building process to assign appropriate names to the subcategories, ensuring that the individual context factors within each subcategory were accurately represented. This synthesis extended beyond simple categorization and subcategorization, emphasizing continuous alignment and agreement among all involved. As a result of this collaborative approach, the context factors were not only precisely defined but also consistently aligned with the context framework established byZimmermann et al. [20]

## Results

**Fig. 1:**
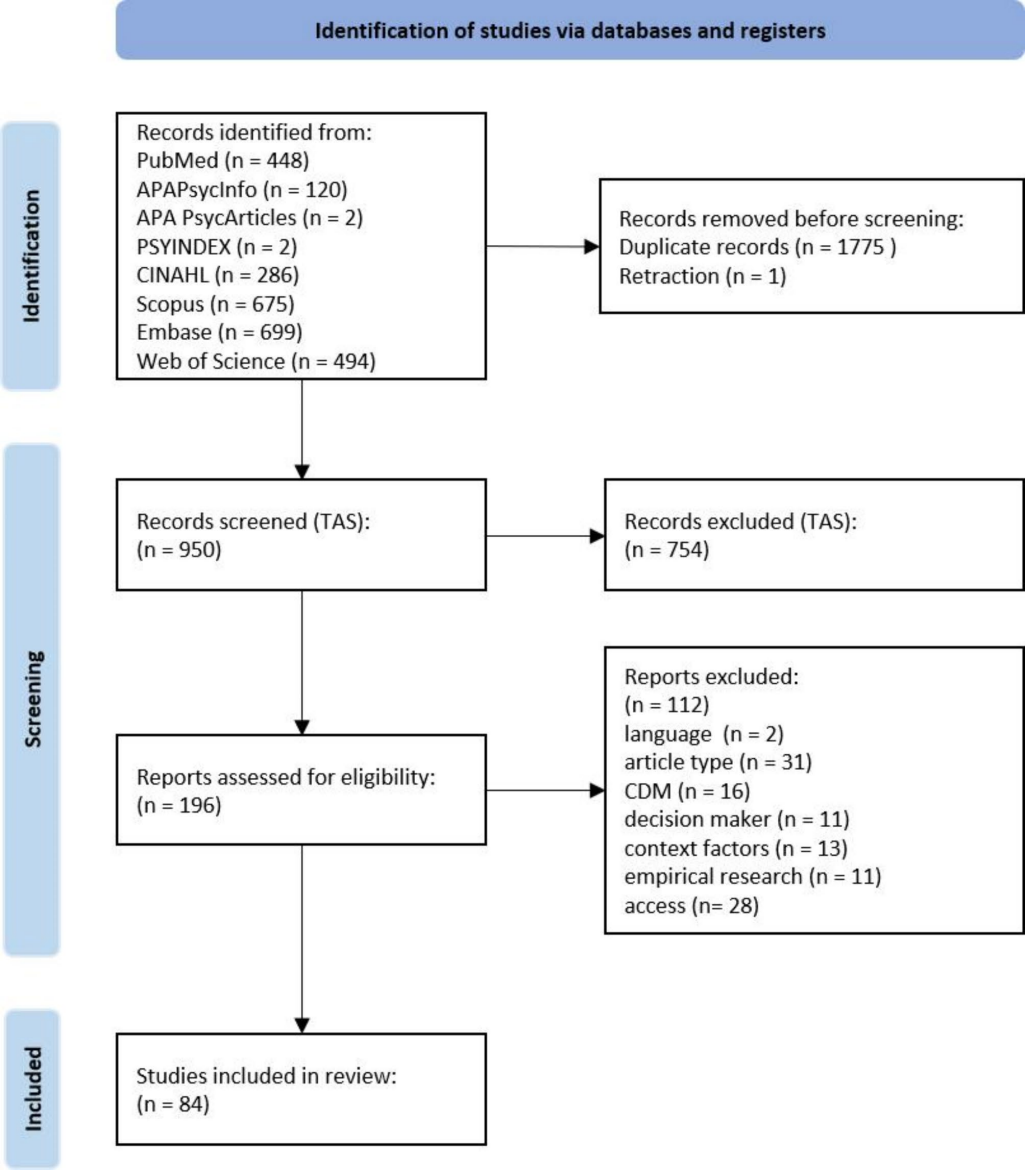
PRISMA flow diagram for the article selection and review. TAS = title abstract screen; FTS = full-text screen; CDM = clinical decision-making

The initial database search identified 2,726 publications. After removing 1,775 duplicates and one retraction, 950 publications were subjected to title and abstract screening (TAS). Following the exclusion of 754 publications during TAS, 196 publications proceeded to the full-text screening (FTS) phase. Applying the predefined exclusion criteria (Table 3), 112 publications were excluded during FTS, resulting in the final inclusion of 84 publications for synthesis. The screening process and its outcomes are illustrated in Fig. 1, following the PRISMA (Preferred Reporting Items for Systematic Reviews and Meta-Analyses) guidelines outlined by Page et al. [44].

### Metadata

Except for one conference paper [45], all included publications are journal articles. The 84 publications included in the review were published between 1990 and 2022. Over half of the publications (55%) were published between 2017 and 2022 (2022: *n* = 12; 2021: *n* = 8; 2020: *n* = 9; 2019: *n* = 7; 2018: *n* = 5; 2017: *n* = 5). The majority of the publications included in the synthesis were published by authors affiliated with institutions from the USA (37%), the United Kingdom (17%), Germany (7%), Canada (6%), and the Netherlands (5%).

### Study characteristics

#### Research type

The studies included in the review (*N* = 84) are exclusively empirical investigations. Among these, 56% were conducted using quantitative research methods, and 31% employed qualitative research methods. The remaining 13% utilized a mixed-methods approach, incorporating convergent parallel design (quantitative and qualitative data collected simultaneously) and explanatory or exploratory sequential design (one type of data collection follows the other).

#### Site of study conduction

The site of study conduction describes the country where the studies included in this review (*N* = 84) were conducted. A total of 31% of the studies were conducted in the USA, while 14% took place in the UK. Germany was the site of data collection for 6% of the studies, with Australia and Canada each accounting for 5%. Other locations appeared only once or twice and are thus not elaborated upon. Notably, 21% of the studies involved multinational sites, meaning data collection occurred in more than one country. For example, the publications by Acem et al. [46] and Nugraha et al. [47] mention continental data collection sites.

### Medical scope

#### Care sector

The studies included in the data synthesis (*N* = 84) were conducted in 45% of cases within the inpatient sector and in 30% of cases within the outpatient sector. Data collection occurred in 20% of the included publications in inpatient and outpatient care settings. For 5% of the publications [48–51], it was impossible to determine the care sector in which the data collection was conducted.

#### Specialty area

The specialty area indicates the medical field in which the studies included in this review were conducted. Context factors were examined across 21 different medical specialties. In nine (11%) publications, no specific specialty was mentioned [45, 50, 52–58], while eight (9%) publications spanned multiple specialties [21, 59–65]. Primary care was most frequently mentioned among the articles that specified a particular specialty (80%; *n* = 67), followed by oncology and internal medicine. Context factors were also examined in emergency medicine, cardiology, surgery, pediatrics, and intensive care. Other specialties, each represented by a maximum of two publications, included nephrology, dermatology, stroke care, psychiatry, trauma care, rehabilitation, psychology, palliative care, orthopedics, general medicine, AIDS care, addiction medicine, and acute care.

#### Clinical decision

The studies included in the data synthesis (*N* = 84) primarily investigated clinical decisions related to interventions, comprising *n* = 45 (54%) of the publications. Additional *n* = 6 (7%) of the studies focused on decision-making processes concerning diagnoses. A total of *n* = 11 (13%) of the studies examined diagnostic and intervention decisions, while *n* = 3 (4%) of the publications concentrated on monitoring decisions. Context factors related to admission decisions were investigated in *n* = 6 (7%) of the included publications. Additionally, *n* = 3 (4%) studies focused on management decisions, and one study investigated contextual factors relevant to both monitoring and intervention decisions. In *n* = 9 (11%) of the publications, the decision-making process was not specified in detail and was broadly referred to as a clinical decision-making process.

### Context factors

A total of 946 context factors were extracted from the included publications (*N* = 84) during the data extraction process. This included both factors that, according to the authors of the publication, influenced clinical decision-making in the respective studies (91%; *n* = 859) and factors for which no influence was reported (9%; *n* = 87). Among the identified context factors (*N* = 946), *n* = 847 (90%) were unique factors, while *n* = 99 (10%) were duplicates mentioned in more than one publication.

#### Entities and context category

**Fig. 2:**
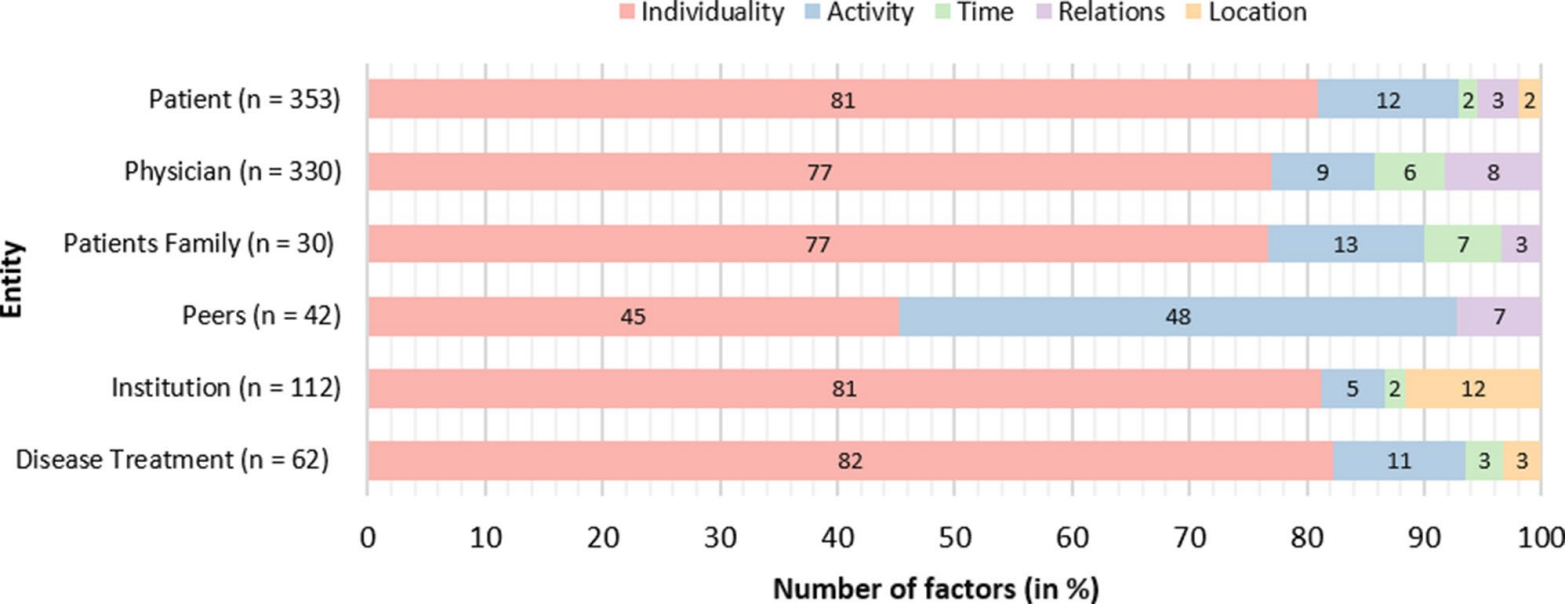
Assigned context factors are per entity and context category

Since the extracted context factors in the included publications were described at different levels of aggregation, such as patient demographics [21, 66, 67] versus patient marital status [68–70], these were categorized and assigned to corresponding entities through consensus using a card sorting method [42]. This step was undertaken to enable an appropriate categorization of the identified factors. Through the card sorting process, a total of six entities were identified, to which the context factors were assigned. These entities include the patient, the physician, the patient’s family, the institution (where the physician operates), the physician’s peers, and the disease treatment. According to Zimmermann et al. [20], the context of each entity can be defined by the fundamental context categories of individuality, activity, relations, time, and location. Consequently, the context factors were further assigned to the relevant context categories of the identified entities and then subdivided into additional subcategories. Of the total *n* = 929 context factors, 38% (*n* = 353) were assigned to the patient, 36% (*n* = 330) to the physician, 12% (*n* = 112) to the institution, 5% (*n* = 42) to the peers, 3% (*n* = 30) to the patient’s family, and 7% (*n* = 62) to the disease treatment. Figure 2 provides an overview of the percentage categorization of factors concerning the context category of the corresponding entities. As illustrated in Fig. 2, individuality has the highest number of context factors for all entities except for peers. For peers, the activity category is particularly prominent, and the patient’s family entity has notably many factors within the location context category compared to the other entities.

The identified context factors for each entitiy were further subdivided into subcategories based on the fundamental context categories. Table 5 provides an overview of the determined subcategories for each entity and their underlying context categories. Additionally, the table lists the relevant publications from which the context factors were extracted.

**Table 5:** Categorization of context factors for identified entities

Entity	Context category	Subcategory	Reference
Patient	Individuality	Patient traits	[52, 71]
		Knowledge	[52, 64, 66, 68, 72–76]
		Experience	[72, 77]
		History	[48, 66, 69, 78–80]
		Social situation	[45, 52, 66, 67, 69, 72, 75, 81–84]
		Health status	[21, 50, 52, 54, 58, 62, 64–67, 69–75, 79–82, 84–103]
		Access	[52, 53, 66, 68, 71, 72, 75, 95, 104]
		Wants & needs	[50, 55, 62, 64–66, 71, 72, 74, 77, 86, 88, 89, 91, 93, 95, 99, 104–111]
		Demographics	[21, 45, 48, 49, 51, 53, 57, 65–72, 79, 80, 82, 84–87, 90–92, 94, 95, 97, 98, 102, 103, 110, 112–119]
		Appearance	[48]
		Personality	[72]
		Psychological factors	[48, 51–54, 64–66, 71, 77, 80, 97, 98, 102, 109, 115, 120]
		Work-related factors	[53, 72]
	Activity	Physical activity	[95]
		Choice of treatment	[65]
		Diagnostic suggestion	[59, 98]
		Behavior	[21, 48, 50, 52, 53, 57, 64, 66, 67, 69, 71–73, 75, 76, 80, 82, 95, 99, 103, 105, 106, 116, 121]
		Medication request	[21]
		Expression of emotions	[69]
	Time	Time limitations	[108]
		Visit time	[21]
		Time till treatment start	[70, 118]
		Admission time	[80]
	Relations	Social environment	[66, 68, 71, 72, 90, 91, 114]
		Health care provider	[21, 68, 72]
		Disease treatment	[72]
	Location	Place of residence	[70, 71]
		Treatment location	[53, 66, 69, 72]
Physician	Individuality	Demographics	[48, 49, 52–54, 58, 61–64, 67, 69, 73, 74, 76, 79, 81–84, 86, 89–94, 96, 103–105, 107, 108, 111–113, 122–125]
		Emotions & feelings	[48, 53, 69, 76, 104–107, 110, 116, 118, 121, 125]
		Health status	[115, 122]
		Wants, needs, opinion	[21, 61, 62, 69, 76, 81, 88, 104, 105, 108, 111, 116, 118, 120, 122, 123, 125, 126]
		Skills & knowledge	[48–50, 52–57, 59, 61–64, 69, 73, 74, 81, 83, 84, 86, 88–91, 96, 99, 101, 103–105, 107–110, 112–116, 120, 122, 124, 125]
		Norms & values	[69, 81, 104, 108]
		Personal states	[21, 53, 56, 57, 65, 71, 81, 89, 101, 104, 108, 113, 115, 118, 121, 125, 127]
		Perception	[104, 110]
		Personal traits	[21, 64, 66, 89, 125, 127]
		Habits	[60, 61, 81, 108, 111]
	Activity	Documentation	[81, 86]
		Treatment	[21, 61, 64, 77, 84, 101, 109, 111, 118, 120]
		Decision making	[59, 61, 65, 66, 81, 86, 89, 90, 101, 104, 107, 110, 127]
	Time	Time of day	[60]
		Amount of time	[21, 45, 53, 56, 61, 64, 76, 77, 81, 93, 98, 113]
		Time management	[50, 76, 86, 99, 109, 126]
	Relations	Patient	[43, 55, 59, 67, 68, 74, 75, 79, 87, 89, 92, 93, 98, 104, 106, 108, 109, 111]
		Industry	[21, 54, 61, 92]
		Colleagues	[78]
Disease treatment	Individuality	Treatment costs	[50, 62, 69, 71, 73, 91, 96, 104, 128]
		Course	[69, 71, 72, 80, 88, 92, 94, 111]
		Complexity	[89, 92, 101, 111]
		Evidence	[49, 74, 88, 96, 105, 118, 125]
		History	[48, 65, 66, 69, 72, 84, 98, 121]
		Severity	[21, 62, 73, 79, 88, 94, 99, 116, 118]
	Activity	Treatment options	[55, 69, 90, 99, 121]
	Time	Treatment time	[57, 86]
	Location	Care factors	[72]
Institution	Individuality	Hospital factors	[21, 45, 54, 55, 61, 62, 65, 66, 69, 71, 73, 75, 77, 80, 83, 85, 86, 88–91, 95, 96, 100, 101, 104, 106, 108–111, 113, 115, 117, 118, 121, 123, 126, 127, 129]
		Culture	[101, 106, 120, 123]
		Guidelines	[50, 54, 62, 63, 83, 88, 95, 101, 103, 109, 113, 120, 123]
		Scope of practice	[81, 86, 89]
	Activity	Management	[69, 86, 89, 90, 101, 113]
	Time	Constraints	[71, 73]
	Location	Geographic	[50, 57, 61, 69, 76, 77, 82, 86, 117, 126]
Family of patient	Individuality	Abilities	[100]
		History	[21, 95]
		Appearance	[124]
		Attitude	[69, 88, 91, 92, 100, 102, 105, 117, 123, 124]
		Living situation	[53, 69]
		Demographics	[72, 123, 124]
		Knowledge	[123]
	Activity	Behavior	[77, 116, 123]
		Decision making	[123]
	Time	Time constraints	[71, 73]
	Relations	Parental absence	[69]
Peers	Individuality	Opinion	[52, 88, 105, 110, 124]
		Perception	[88]
		Involvement	[65, 70]
		Experience	[62, 124–126]
		Constraints	[73, 108]
		Team structure	[74, 86, 104, 120]
	Activity	Decision making	[69, 101, 126]
		Behavior	[54, 61, 64, 74, 86, 88, 89, 105, 121]
		Interaction	[69, 74, 76, 77, 86, 109]
	Relations	Professionals	[121]
		Hospital staff	[52, 113]

#### Patient-related context factors

The majority of context factors related to the patient (*n* = 353) are associated with individual characteristics and attributes of the patient (*n* = 286; 81%), with 92% of the individual factors reported as influencing the clinical decision-making process. In terms of individuality factors, the most frequently described context factors (33%) pertain to the patient’s health status, including treatment-related attributes, for example, treatment success [21, 64, 69, 73, 90, 91], medication [70, 91, 95], treatment compliance [84], provision of consent [89], performance status [64, 87, 91], weight [50, 68, 69, 73], risk factors [79, 89, 92, 95, 100, 109], or comorbidities [50, 52, 62, 65, 67, 72, 80, 82, 85, 87, 89, 91, 95, 99, 107], as well as symptom-related factors (e.g., number of symptoms [94], quality of life [69, 71, 96, 99], and diagnostically relevant context factors such as obesity [67, 95], alcoholism [52, 67, 73] or psychiatric conditions [52, 123]. Demographic data of the patient were frequently mentioned (28%) in terms of individual characteristics, including age [45, 48, 49, 57, 69–72, 82, 84–87, 90–92, 94, 95, 99, 102, 103, 110, 112, 114, 116–118], gender [45, 49, 70–72, 79, 80, 94, 114, 116, 117], religion [68–70], marital status [72, 125], insurance [66, 82, 113], education [51, 53, 66, 71, 72, 97, 98, 115], culture [45, 48, 68, 69, 71, 72, 79, 84, 95, 118, 119], and the patient’s socioeconomic status [48, 53, 65, 68, 71, 72, 112, 114, 119]. Additionally, the wants and needs of a patient, including preferences [50, 72, 74, 95, 99, 104, 105, 110, 111], expectations [71, 72, 93, 106, 107, 109], wishes [55, 62, 65], and opinions [91] or beliefs [108], were reported (10%). Psychological factors, such as attitude [68, 71, 109], concerns [53, 66, 72, 75, 101], and emotions and feelings (e.g., emotional volatility or hopelessness), were also reported in 9% as context factors influencing clinical decisions [51, 64, 68, 72, 77, 80, 97, 98, 102, 115]. Concerning activity-related factors (*n* = 42; 12%) of the patient, which were reported as influencing the clinical decision-making process in 93% of cases, patient behavior was cited (85%) as an influencing factor, including adherence [48, 50, 66, 71, 72, 75, 76, 82, 95, 116] or general compliance of the patient [80, 105, 116], as well as abusive behavior characteristics such as drug abuse like smoking [57, 72, 95, 103] or alcohol intake [95]. Context factors related to the patient’s relationships accounted for 3% (*n* = 12) of all patient-related factors and were reported as influencing the clinical decision-making process. It was noted that the social environment in particular, such as relationships with family [71, 90] and relationship with healthcare providers [21, 68, 72] have an impact on clinical decisions. Location-related (*n* = 7; 2%) and time-related (*n* = 6; 2%) factors were also reported as influencing factors. For time-related factors, examples include the duration of a patient’s hospital stay [21] or the time to treatment initiation (e.g., time from diagnosis to treatment [70]). For location-related attributes, the patient’s place of residence (e.g., distance from home to hospital [70]) and the treatment location (e.g., inpatient vs. outpatient [53, 66]) were described as contextual influencing factors.

#### Physician-related context factors

Similar to the patient-related factors, most factors concerning the physician (*n* = 330) are related to individual characteristics (*n* = 254; 77%), with 84% of the individual factors described as influencing factors. The most frequently described (30%) were demographic characteristics, such as age [48, 49, 61, 62, 84, 103, 107, 122, 124], gender [52, 53, 58, 62, 63, 76, 84, 89, 91, 93, 107, 108, 112, 122, 124], profession [52, 58, 61, 63, 64, 67, 73, 74, 79, 81–83, 86, 89, 90, 92, 94, 96, 105, 111, 113, 122, 123, 125], education [54, 63, 90], religion [90, 107, 122], or the physician’s position [64, 69, 83, 107, 113]. Factors related to the subcategory of skills and knowledge were also frequently described (26%), with knowledge itself being a commonly mentioned factor [61, 64, 74, 81, 88, 90, 91, 104, 108, 109, 125] along with experience [49, 53, 54, 56, 57, 59, 61–64, 69, 73, 83, 88–90, 96, 103–105, 110, 112, 114–116, 120, 124, 125], expertise [74, 99, 113], or training [48, 53, 56, 61, 83, 89, 105]. Factors encompassing the wants and needs of the physician, such as preferences [104, 105, 120, 126], wishes [69], attitude [21, 61, 81, 88, 104, 105, 108, 116, 118, 122, 123, 125], expectations [76, 104], opinion [21, 108, 111], and interests [62, 108], accounted for 17% of the identified individual factors. Another 10% of the physician’s individuality factors pertain to personal states, including awareness [21, 81, 104, 108, 125], workload [53, 56, 57, 71, 101, 113, 118, 121, 127], stress (both mental and physical) [53, 101, 113], sleepiness [115], time pressure [65, 101], and confidence [89, 105]. An additional 7% relate to the emotions and feelings of the physician, such as fear [121], trust [48, 105, 107], or comfort [110, 118]. Activity-related factors (*n* = 29; 9%) were reported as influencing factors by the authors of the included publications, with one exception. These factors primarily pertain to treatment-specific activities (45%), such as interaction and communication with the patient [21, 61, 64, 101, 109, 111, 118, 120]. Another 45% are directly related to decision-making, such as biases (e.g., tunneling, ascertainment [81, 89], type of decision (ordering vs. prescribing) [107], cost considerations [104], or inadequate information [65, 127]. The remaining activity-related factors are associated with documentation activities (e.g., reporting outcomes) [81, 86]. Context factors related to the physician’s relationships (*n* = 27; 8%) were also predominantly reported as influencing factors. The majority (81%) pertain to the physician-patient relationship, including factors related to interaction with the patient [21, 69, 101, 104, 116, 121, 123], appointment-related factors such as a new appointment [53], perceived compliance [79], and perceived risks and benefits of therapy, treatment or interventions [77, 78, 87, 96, 99, 105, 110, 118, 120, 123]. Time-related context factors (*n* = 20; 6%) concerning the physician were exclusively reported as influencing the clinical decision-making process. The most frequently mentioned factors (65%) were related to the amount of time available to the physician, such as lack of time [21, 76, 77, 113], time limits [61], rushed visits [81], or time spent with the patient [64]. Another 30% describe the physician’s time management, such as time schedules [86] or waiting time [126] (e.g., for an urgent outpatient appointment [76]). All location-related factors concerning the physician are associated with the institution where the physician operates, defined as a separate entity, and will be described in further detail.

#### Institution-related context factors

The total of *n* = 112 identified context factors related to the institution (where the treating physician operates) are predominantly associated with individual characteristics of the institution (*n* = 91; 81%), with 95% of the individual factors reported as influencing the clinical decision-making process. Most of the individuality characteristics (76%) can be grouped under hospital factors and pertain to hospital structure, such as the practice type (private vs. non-private) [71, 104, 108, 109], clinical standards [62, 90], or the size of the organization [86]. Additionally, legal and finance factors, such as financial constraints [73, 88], clinical load [21, 101, 118, 127], clinical improvement [80, 100], clinical load [21, 101, 118] (e.g. overcrowding in inpatient units [127]), available resources [61, 65, 66, 71, 75, 77, 86, 88, 95, 96, 100, 101, 106, 109, 110, 117, 121, 123, 125, 126] (such as technical infrastructure [65, 75, 86, 110, 125, 126] or treatment and therapy options [71, 96, 109, 117]), as well as provider continuity [66], were frequently mentioned. While the availability of technical resources was identified as an influencing factor in the reviewed articles, no specific context factors directly pertaining to the system level could be determined. Furthermore, guidelines were frequently described as influencing individual characteristics (16%), including clinical and professional organizational guidelines [50, 54, 62, 63, 83, 88, 95, 101, 103, 109, 113, 120, 123]. Factors related to the culture of the institution, such as prescribing culture [120] or ethical challenges [101] and the scope of practice [81, 86, 89], can also influence the clinical decision-making process. Activity-related factors of the organization (*n* = 6; 5%) were all reported as influencing factors and primarily concerned the management of the institution, such as resource management [89] or operational management [113]. Regarding time-related factors (*n* = 2; 2%), time constraints [71, 73] were reported as contextual influences. Location-related factors (*n* = 13; 12%) were described as influencing in 85% of cases and relate to the geographical characteristics of the institution, including country [54], workplace [61, 77], or practice type (rural or provincial) [76]. No relationship factors were explicitly described in the literature; however, the institution inherently maintains a direct connection with the practicing physician, implying an implicit relationship with the physician.

#### Peers-related context factors

Regarding context factors related to peers (*n* = 42), the majority (48%) pertain to activity-related factors (*n* = 20; 48%), which were all reported as influencing the clinical decision-making process. These activity-related context factors include behavior-related factors (45%), such as communication-related aspects (e.g., team communication [86]) or support from colleagues [89, 105, 121]. Additionally, 30% of activity-related context factors of peers describe interaction-related aspects, such as interdisciplinary ways of working [77, 86]. Overall, 25% of activity context factors describe aspects related to the decision-making of peers (e.g., differences in team members’ decision criteria [101]). Regarding individuality factors (*n* = 19; 45%), 84% were reported as influencing the clinical decision-making process. Most of these factors pertain to peers’ opinions (26%) [88, 91, 105, 110, 124] or peers’ experience (26%) [62, 124–126]. Other factors include aspects of team structure [74, 86, 104, 120], perception [88], constraints [73, 108], and the degree of involvement of peers [65, 70]. For relation-related factors (*n* = 3; 7%), the relationships between peers and other professionals [121] and with hospital staff [52, 113] were reported as influencing context factors in the clinical decision-making process. Location-related and time-related context factors concerning peers were not identified in the included publications.

#### Family of patient-related context factors

A total of *n* = 30 context factors were assigned to the patient’s family, with the majority (*n* = 23; 77%) pertaining to individual characteristics of the family. Of these individual factors, 87% were reported as influencing clinical decisions. Most frequently (52%), aspects related to the family’s attitude were mentioned, such as family preferences [102, 105, 117], parents’ perception [88], opinion [91], or concerns [69]. Other individual characteristics of the patient’s family included abilities [100], family history [21, 95], appearances [124], living situation [53, 69], demographics [72, 123, 124], and knowledge-related factors such as the understanding of futility of treatment [123]. Regarding activity-related factors (*n* = 4; 13%), behaviors like family support [116] or encouragement [77], as well as family decisions [123], were reported as influencing the clinical decision-making process. Similarly, time-related factors (*n* = 2; 7%) related to the family’s constraints [71, 73] were identified as influential. In terms of relation factors (*n* = 1; 3%), parental absence [69] was reported as an influencing context factor in the clinical decision-making process. Location-related context factors concerning the family were not identified in the scope of this work.

#### Disease treatment-related context factors

A total of *n* = 62 context factors related to disease treatment were identified in the included publications. Of these, *n* = 51 (82%) are individuality characteristics, reported as influencing the clinical decision-making process in 96% of cases. These factors pertain to treatment costs [50, 62, 69, 71, 73, 91, 96, 104, 128], course of disease [69, 71, 72, 80, 88, 92, 94, 111], overall complexity of the disease [89, 92, 101, 111], existing evidence base [49, 74, 88, 96, 105, 118, 125], treatment history [48, 66, 69, 74, 84, 98, 121], and severity of disease [21, 62, 73, 79, 88, 94, 99, 116, 118]. An additional *n* = 7 (11%) context factors, all reported as influencing factors, pertaining to the activity characteristics of disease treatment and can be grouped under the category of treatment options, such as treatment options [99], treatment alternatives [121], or organ donation [69]. Regarding time-related characteristics (*n* = 2; 3%), the treatment period [86] and time elapsed since diagnosis [57] were reported as influencing factors. For location-related characteristics (*n* = 2; 3%), care factors such as the functionality of informal care [72] and functionality of formal care [72] were identified as influencing the clinical decision-making process. Relationship-related context factors concerning disease treatment were not identified within the scope of this work.

### Quality assessment

A comprehensive total score was calculated to assess the overall methodological quality of the included studies (*n* = 84). Initially, the mean score of the ratings from both reviewers was determined separately for each assessment item. Subsequently, the sum of these mean scores was calculated and divided by the number of items, yielding the average rating across all items for both reviewers. The resulting total score across all studies was M = 3.10 (SD = 0.51), indicating that the methodological quality of the included studies is rated as moderate.

## Discussion

This review provides a comprehensive overview of the contextual factors previously investigated in physicians’ clinical decision-making process. The central focus was to assign the identified contextual factors to corresponding entities and subsequently categorize them into an applicable structure. A literature search across nine different databases identified 84 articles, from which 946 contextual factors were extracted. These factors were categorized using the card sorting methodology into the main categories of individuality, activity, time, location, and relation, and were assigned to the entities of patient, physician, patient’s family, peers, institution, and disease treatment. The majority of the contextual factors were associated with the individual characteristics and attributes of the entities, with most factors identified in both the patient and the physician.

Patient-related contextual factors primarily described individual attributes such as health status (e.g., treatment success, medication adherence, or comorbidities) or patient demographic data (e.g., age, gender, or socioeconomic status). Factors identified concerning physicians also predominantly described individual characteristics, including demographic data and skills of the physician (e.g., knowledge, clinical experience, or expertise). Furthermore, contextual factors related to disease treatment were identified, such as treatment costs, disease progression, or severity of the illness, as well as institutional factors encompassing aspects such as hospital structure, institutional resources, and clinical guidelines. Additional contextual factors pertained to peers, including team communication and interdisciplinary collaboration, as well as to the patient’s family, particularly their attitudes, support structures, and living situations. While we acknowledge that certain context factors may vary in relevance depending on the medical specialty or use case (e.g., oncology, emergency medicine), our focus was to identify general context factors that could be applicable across a range of clinical settings. We emphasize that not all context factors are universally relevant for all specialties or applications, and the importance of these factors may differ significantly depending on the specific context of use.

The findings of this work highlight the complexity of the construct of context and demonstrate the variety of factors that can influence the clinical decision-making process. To the best of our knowledge, this work is the most comprehensive and systematic summary of contextual factors influencing the clinical decision-making process published to date. This underscores the necessity of adequately considering contextual factors in the clinical decision-making process, as these can significantly impact patient care. In particular, accounting for the specific conditions of disease treatment, such as severity, complexity, and progression, is essential for optimizing treatment to fit each patient’s individual needs. Moreover, patient-related factors play a crucial role in personalized care, as they offer a comprehensive view of the patient’s health status, facilitating the development of tailored prevention strategies and treatment plans that address the unique needs and conditions of the patient [130]. Clinical decision support systems (CDSS) can assist physicians in capturing these patient-specific factors and integrating them into the decision-making process [4, 9]. It is crucial to consider not only patient-centered factors but also familial influence factors. Knowledge of family medical history allows for the early identification of genetic risks and the preventive adjustment of treatment plans [131]. A context-sensitive CDSS that considers familial health risks and support structures can help physicians create patient-centered treatment plans while promoting family involvement.

Considering physician-related contextual factors is also essential to technologically support clinical decision-making effectively. Inadequate experience, lack of knowledge, and high workload, fatigue, or stress on the part of the physician can lead to misdiagnoses and erroneous treatments [132]. Regular training courses and simulation training can help physicians improve their diagnostic and therapeutic skills [133]. Technology-based systems, such as CDSS, can support workflow efficiency [9] by automating routine tasks like patient data documentation and report generation, thereby reducing workload and associated stress [134]. Moreover, CDSS can incorporate the expertise and preferences of physicians through learning algorithms, allowing for the personalization of decision paths [135].

The quality of clinical decisions can also be enhanced by support from experienced colleagues and collaborative approaches [136, 137]. CDSS can support collaboration between the treating physician and peers by facilitating access to relevant information and communication among team members [138]. However, this requires considering institutional conditions such as resource availability, work environment, and institutional guidelines. A lack of sufficient resources, such as staff or medical equipment, can lead to delayed diagnoses and treatments, ultimately impacting patient care [139]. Successful implementation of CDSS is significantly dependent on adaptation to the specific needs and conditions of the institution, as inadequate adaptation of guidelines to local circumstances can ultimately reduce system acceptance [139].

CDSS have significant potential to enhance patient-centered care by supporting medical decisions with tailored clinical information, while simultaneously addressing patients’ individual needs and preferences. By considering contextual factors such as socioeconomic background, individual health goals, and family circumstances, CDSS facilitate personalized decision-making focusing more on the patient. Integrating the Patient and Public Involvement (PPI) approach into the development of CDSS can ensure that patient perspectives and needs are systematically considered from the outset. This approach increases the context sensitivity of the systems by incorporating factors such as socioeconomic backgrounds and family circumstances into decision-making processes early on. As a result, both the relevance and effectiveness of CDSS are improved, ultimately leading to even more patient-centered care. Additionally, the consideration of contextual factors in CDSS contributes significantly to achieving the Sustainable Development Goals (SDGs) [140], particularly SDG 3 (Good Health and Well-being) [141]. By accounting for local circumstances (e.g., cultural and socioeconomic factors) and adapting to individual needs and regional resources, the quality of care can be substantially improved, contributing to the reduction of health disparities.

Although this review provides a comprehensive overview, it may not identify all contextual factors discussed in the literature due to the specific search strategy used. The focus on published peer-reviewed articles and primary research studies excluded insights from gray literature, such as dissertations and government documents, as well as review articles. Additionally, the search was conducted in April 2023, and no updated or backward citation searches were performed, which may limit the comprehensiveness of the findings. However, the large number of identified contextual factors and the occurrence of duplicates of specific factors can indicate that the study has already captured the most relevant factors. Another area for improvement is the heterogeneity of the included studies, which were conducted in different medical settings and levels of care, making it challenging to make statements regarding the transferability of the reported contextual factors. Furthermore, this work does not allow for conclusions regarding the extent and strength of the influence of the examined contextual factors on the clinical decision-making process. Similarly, potential interactions between the factors were not investigated, preventing conclusions about possible moderator effects. Moreover, this study exclusively examined the decision-making process of physicians. This focus was chosen because physicians’ decision-making process fundamentally differs from that of other healthcare professionals, such as nurses [142, 143], with these differences being considered both necessary and complementary [144]. While physicians primarily focus on diagnoses and treatment plans, emphasizing medical issues and causal relationships, nurses tend to focus more on patient care needs and the patient’s social environment. Furthermore, nurses work more frequently in interdisciplinary teams, incorporating the patient’s social context into their decision-making [143, 144].

Given the increasing use of interprofessional teams in healthcare, it is essential to examine the interplay between individual and interprofessional decision-making and the influence of contextual factors. Addressing these dynamics is crucial to designing CDSS that effectively support both decision-making processes. Future research should focus on empirically investigating the transferability of the identified factors across different medical specialties and levels of care. Furthermore, the generalizability of contextual factors across various disciplines, decision-making scenarios, and clinical conditions should be systematically analyzed. It is also important to examine potential interactions between these factors to validate and expand their relevance and applicability in different medical contexts. This would enhance the transferability of findings and increase their practical utility for clinical decision-making. These aspects should therefore be key priorities for future research.

The present study focused on compiling and categorizing previously examined context factors. However, the targeted integration of these factors into clinical decision support systems (CDSS) lacks specific methodological approaches that would enable developers to systematically capture, prioritize, and implement them. A key approach in this regard is the User-Centered Design (UCD) process [12, 13], in which context of use analysis plays a central role. As part of this analysis, relevant conditions, influencing factors, and user needs are systematically examined and identified to develop a comprehensive understanding of the usage context and establish well-founded requirements for system development or optimization [145]. While established UCD methods such as interviews and observations provide valuable insights into the usage context, a structured approach for the systematic identification of contextual influencing factors for CDSS remains lacking. Future research should develop targeted methodological frameworks to systematically capture these factors and facilitate their structured integration into CDSS. It is also essential to consider system-specific factors such as interoperability and reliability, as these are critical for the successful implementation of context-sensitive CDSS. This study provides an initial foundation for identifying relevant factors and developing practical assessment and implementation frameworks that support patient-centered decision-making.

Furthermore, the findings of this study offer important implications for practice, education, and policy. In practice, context-sensitive CDSS can improve physicians’ decision-making processes by considering individual patient characteristics, institutional conditions, and the influence of family and colleagues to provide targeted recommendations. In education, healthcare curricula should emphasize the importance the importance of contextual factors and interdisciplinary collaboration to better prepare future professionals for complex decision-making situations. From a policy perspective, decisions should support the development and implementation of CDSS to enhance patient-centered care and improve the overall quality of healthcare.

## Conclusion

The study highlights the complexity of the context construct in clinical decision-making and emphasizes the necessity for systematic collection and consideration of contextual influence factors. The literature analysis demonstrated that the characteristics of the patient, the patient’s family, the institution, disease treatment, peers, and the physician themselves are influential factors in the clinical decision-making process. Adapting CDSS to the contextual conditions in which clinical decisions are made is a fundamental requirement for these systems to provide personalized and precise decisions. Based on the study results, the need for a comprehensive and systematic operationalization of the identified contextual factors becomes evident. This is a prerequisite for developing suitable concepts to integrate these factors into CDSS, supporting personalized and optimized patient care. By systematically compiling and categorizing these factors, this study provides a foundational framework that facilitates the structured integration of contextual influences into CDSS, enhancing their relevance and effectiveness in real-world clinical settings. Addressing these factors in CDSS development can improve decision-making processes by incorporating individual patient characteristics, institutional conditions, and interprofessional collaboration, ultimately supporting patient-centered and evidence-based care. The findings also have significant implications for practice, education, and policy. In practice, context-sensitive CDSS can enhance decision-making by incorporating patient characteristics and institutional conditions, improving diagnostic accuracy and treatment outcomes. Healthcare institutions should prioritize their integration into clinical workflows to optimize efficiency. At the same time, policy initiatives should support the development and implementation of such systems by promoting interoperability, data security, and equitable access. In education, medical training should emphasize the role of contextual factors in decision-making and equip clinicians with the skills to effectively utilize CDSS, fostering evidence-based and patient-centered care.

## Data Availability

Data is provided within the manuscript.
